# Shear band analysis based on a strain-gradient hypoplastic model

**DOI:** 10.1007/s11440-026-02956-0

**Published:** 2026-03-16

**Authors:** Xuan Kang, Wei Wu, Ivan Zaboev, Xiaogang Guo

**Affiliations:** Institut für Geotechnik, Universität für Bodenkultur Wien, Feistmantelstraße 4, A-1180 Vienna, Austria

**Keywords:** Hypoplastic model, Post-localization regime, Shear band thickness, Simple shear test, Strain gradient

## Abstract

Localized deformation is commonly observed in granular materials, yet it remains significant challenges for constitutive modeling and numerical simulation. This study extends a hypoplastic model for shear band analysis in granular materials by incorporating a second-order gradient of the strain rate into its nonlinear tensorial function. The extended model keeps the conciseness of hypoplastic framework while introducing a length scale to capture the finite shear band thickness in the post-localization regime. This leads to improvements over the original non-gradient model in shear band analysis. Numerical simulations of simple shear tests demonstrate the model’s capability to reproduce finite-thickness shear bands in granular materials with inhomogeneous initial void ratio distributions. Compared to the existing gradient models, the extended model shows superior performance in analyzing realistic shear band problems with rotation.

## Introduction

Granular materials composed of numerous discrete particles can exhibit complex behaviors under external loading. They behave like solids at rest and transition to fluid-like behavior when flowing. Although granular materials are inherently discrete, they are often modeled as continua for practical analysis. However, this simplification gives rise to challenges in capturing highly localized deformation, such as shear band formation. While the thickness of a shear band is comparable to the particle size or microstructural features, the granular material show scale-dependent behavior and cannot be fully addressed by conventional continuum theories, which lack inherent length scales [7, 24]. Such problems are commonly observed in micro-mechanics [21], high-rate deformation [22], and phase transition, e.g., granular materials transfer from solid to flow behavior [5, 11].

To address the limitations in shear band analysis, several enhanced constitutive frameworks have been developed, such as strain-gradient models [1], micropolar models [17, 20, 25], and non-local models [6, 18]. These approaches introduce inherent length scales into the formulation to consider the essential scale effects like thickness and spacing of shear bands. Unlike classical models, their numerical solutions are free from spurious dependence on the mesh size, enabling more physically meaning of localized deformation. Moreover, these enhanced models provide the linkage between microscopic mechanisms and macroscopic phenomena.

Gradient model was first introduced in plasticity theory to account for localization phenomena at both microstructural and macroscopic scales [1, 10]. It was achieved by introducing the higher-order gradient of the plastic strain into the yield function or the dilatancy formulation without additional stress or strain variables [28, 29]. The boundary conditions related to the gradient terms can be derived using the principle of virtual work [12]. Owing to these merits, a series of gradient models were developed based on elasticity and plasticity to deal with practical problems such as crack tips [19, 35], shear band width [4, 44] and size effects [3, 43]. The applications of such high-order theories in constitutive relations receive discussions, especially in small-strain problems, e.g., shear band analysis in granular materials.

As an alternative to classical plasticity, hypoplastic models offers the ability to describe an elastic phenomena without relying on yield surfaces, plastic potentials, and decomposition. These concepts are inherent byproducts of the constitutive equations. Hypoplastic models demonstrate predictive capabilities comparable to conventional elasto-plastic frameworks [14, 33, 38]. Despite their simplicity, early versions of hypoplastic models were able to capture salient features of granular materials, such as sands and gravels, using only four model parameters [39]. In order to solve the problems with shear band localization at small strains, enhancements of the hypoplastic models have been developed along the lines of the micropolar theories [15, 26] and the non-local theories [18, 36].

In this study, an idea proposed by Wu [41] of higher-order hypoplastic model is developed. A second-order gradient of the strain rate is introduced into the nonlinear tensorial function of a recently proposed hypoplastic model for granular materials [34]. The extended hypoplastic model captures the strain softening behavior induced by different initial void ratios under inhomogeneous loading conditions. This allows us to describe the deformation process up to the onset of strain localization. Under simple shear conditions, inhomogeneous deformation in the post-localization regime including the evolution of a finite-thickness shear band is well described by the extended model. The formulation of the extended model remains concise and requires only a single material length scale parameter for shear band analysis.

## Hypoplastic framework

To gain perspective, we start with the following hypoplastic constitutive framework proposed by Wu and Kolymbas [37]:

1 $$\begin{aligned} \mathring{\boldsymbol{\sigma }}= {\mathcal {L}}(\boldsymbol{\sigma } ):\dot{\boldsymbol{\epsilon }} + {{\textbf {N}}}(\boldsymbol{\sigma })\left\| \dot{\boldsymbol{\epsilon }} \right\| \end{aligned}$$

where $\boldsymbol{\sigma }$ is the Cauchy stress tensor; $\dot{\boldsymbol{\epsilon }}$ is the strain rate (stretching) tensor. The tensorial functions ${\mathcal {L}}$ and ${{\textbf {N}}}$ are of the 4th and 2nd order, respectively. The colon ${\textbf {:}}$ denotes an inner product between two tensors. $\left\| \dot{\boldsymbol{\epsilon }} \right\|$ stands for the Euclidean norm of the stretching tensor. The Jaumann stress rate tensor $\mathring{\boldsymbol{\sigma }}$ is defined in terms of the material time-derivative of the Cauchy stress tensor $\boldsymbol{\sigma }$ and the spin tensor $\dot{\bf{\omega}}$:

2 $$\begin{aligned} \mathring{\boldsymbol{\sigma }} = \dot{\boldsymbol{\sigma }} + \boldsymbol{\sigma } \dot{\boldsymbol{\omega}} - \dot{\boldsymbol{\omega}} \boldsymbol{\sigma } \end{aligned}$$

The above stretching and spin tensors are related to the velocity gradient tensor through

3 $$\begin{aligned} \dot{\boldsymbol{\epsilon }}= \frac{1}{2}(\triangledown {{{\textbf {v}}}}+{{{\textbf {v}}}}\triangledown ), \quad \dot{\bf{\omega}}= \frac{1}{2}(\triangledown {{{\textbf {v}}}}-{{{\textbf {v}}}}\triangledown ) \end{aligned}$$

where ${{{\textbf {v}}}}$ is the velocity and $\triangledown$ is the gradient operator.

## Strain gradient extension

### Gradient model

Previous shear band analysis based on local models has limitations on scale effect owing to the discrete nature of granular materials. In other words, the thickness of shear bands in the post-localization regime cannot be obtained or depends on the mesh size. While strain-gradient theory provides another approach to achieving an internal length scale. Compared to other continuum models like Cosserat theory, it requires less parameters, which maintain the original intention of the hypoplastic model [41], i.e., concise and precise. In gradient plasticity theory, the yield function [2] and dilatancy function [29] are assumed to depend on the plastic strain and its gradient. In hypoplasticity, the concepts of yield function, flow rule and the decomposition of deformation into elastic and plastic parts from plasticity theory are not used. As shown in Eq. (1), strain is represented by strain rate in hypoplastic model. Therefore, the strain rate gradient instead of the strain gradient is adopted in this study.

The principle of local actions, where deformation at one point only affects material behavior in an infinitely small vicinity [27], is not suitable for heterogeneous deformation with considering averaged deformation due to the material interaction. Nevertheless, the average of deformation does not necessarily have to be applied to the whole constitutive equation. The non-local theories of plasticity are often obtained by assuming local elasticity and non-local plasticity.

The framework given by Eq. (1) shows hypoplastic model contains a linear and nonlinear tensorial function, and the latter depends on the strain rate through its norm. Hereby, Eq. (1) is modified by assuming that the nonlinear part depends on the averaged stretching norm for heterogeneous deformation.

According to Wu [41], the averaged stretching norm can be obtained by looking at the Taylor expansion of $\left\| \dot{\boldsymbol{\epsilon }} \right\|$ in the vicinity of ${{{\textbf {x}}}}$:

4 $$\begin{aligned} \Vert \dot{\boldsymbol{\epsilon }}\Vert ({\textbf{x}} + \Delta {\textbf{x}}) = \Vert \dot{\boldsymbol{\epsilon }}\Vert ({\textbf{x}}) + \nabla \Vert \dot{\boldsymbol{\epsilon }}\Vert : \Delta {\textbf{x}} + \frac{1}{2!} \nabla (\nabla \Vert \dot{\boldsymbol{\epsilon }}\Vert ) : (\Delta {\textbf{x}} \otimes \Delta {\textbf{x}}) + \cdots \end{aligned}$$

where $\nabla$ is the Nabla operator and $\otimes$ denotes a tensor product. Here, $\Delta {\textbf{x}}$ represents the position increment from point ${\textbf{x}}$ to a neighboring point within the representative volume. The above expression can be integrated over a sphere with the radius $R = \Vert \Delta {\textbf{x}}\Vert$ to give the averaged stretching norm. The following expression can be obtained by retaining terms up to the second order

5 $$\begin{aligned} \widehat{\Vert \dot{\boldsymbol{\epsilon }}\Vert } \approx \Vert \dot{\boldsymbol{\epsilon }}\Vert + \frac{1}{V_s} \cdot \frac{2\pi R^5}{15} \nabla ^2 \Vert \dot{\boldsymbol{\epsilon }}\Vert \end{aligned}$$

where $\widehat{\Vert \dot{\boldsymbol{\epsilon }}\Vert }$ is the averaged stretching norm and $V_s$ is the volume of the sphere. Inserting $V_s = (4/3)\pi R^3$ into the above expression leads to

6 $$\begin{aligned} \widehat{\Vert \dot{\boldsymbol{\epsilon }}\Vert } \approx \Vert \dot{\boldsymbol{\epsilon }}\Vert + \frac{R^2}{10} \nabla ^2 \Vert \dot{\boldsymbol{\epsilon }}\Vert \end{aligned}$$

Replacing the local stretching norm in Eq. (1) by the averaged stretching norm and denoting $\lambda ^2 = R^2/10$, we arrive at the following constitutive equation:

7 $$\begin{aligned} \mathring{\boldsymbol{\sigma }}= {\mathcal {L}}(\boldsymbol{\sigma } ):\dot{\boldsymbol{\epsilon }} + {{\textbf {N}}}(\boldsymbol{\sigma })(\left\| \dot{\boldsymbol{\epsilon }} \right\| + \lambda ^2\nabla ^2 \left\| \dot{\boldsymbol{\epsilon }} \right\| ) \end{aligned}$$

Therefore, the material parameter $\lambda$ introduces a material length scale into the constitutive model for governing the influences of strain-gradient effects. Physically, $\lambda$ reflects a microstructural length of the granular material. In numerical implementations, this length scale parameter is often related to material properties such as particle size and shear modulus [45].

### Inherent length analysis

The approach used in elasto-plastic models [24, 29] is adopted here to study the inherent length analysis for the hypoplastic model. We assume a one-dimensional (1D), spatially periodic velocity field imposed on a homogeneous deformation state. The Cartesian coordinates $x_1$, $x_2$, $x_3$ is introduced and a sinusoidal velocity field depending on $x_1$ is given as

8 $$\begin{aligned} v_i(x_1) = a_i \sin (\xi x_1), \quad i= 1,2,3, \quad \xi = \frac{2\pi }{l} \end{aligned}$$

where $a_i$ and *l* represents the amplitude and wavelength of the velocity perturbation, respectively. In the context of gradient-enhanced constitutive models, we examine spatially periodic velocity fields given in Eq. (8) that preserve quasi-static stress equilibrium over an infinitesimal time increment.

This approach establishes a foundation for evaluating the existence of non-trivial solutions, thereby enabling the determination of the material’s inherent length scale. Specifically, if the governing equations admit a solution for a given stress state, the associated wavenumber $\xi$ defines the inherent length *l* pertaining to this state. If periodic solutions are admitted in regions of the stress–strain curve corresponding to hardening behavior, the resulting displacement or velocity fields exhibit unphysical spatial oscillations. These spurious modes indicate a loss of mathematical well-posedness and undermine the predictive capability of the model. Therefore, in a physically consistent formulation, such solutions should arise exclusively in post-localization regimes, i.e., after the peak stress, where the classical (non-gradient) theory predicts the onset of localization. This restriction ensures that gradient terms act as regularizing mechanisms, permitting localized deformation patterns such as shear bands only when they are mechanically justified.

Instead of a continuous stress equilibrium for the velocity field in Eq. (8), the condition of constant stress ($\dot{\boldsymbol{\sigma }}_{1i} = 0$, *i*= 1, 2, 3) is used. Equation (7) with velocity components $v_i(x_i)$ under condition $\dot{\boldsymbol{\sigma }}_{1i} = 0$ can be written as

9 $$\begin{aligned} \eta _{ij} \frac{d v_j}{d x_1} + b_i ||\dot{\boldsymbol{\epsilon }}||+ \lambda ^2 b_i \frac{d^2}{d x_1^2} ||\dot{\boldsymbol{\epsilon }}|| = 0, \quad i = 1, 2, 3 \end{aligned}$$

where $\eta _{ij}$, $b_i$ are functions of the stress components and the void ratio, respectively.

Differentiating Eq. (8) leads to

10 $$\begin{aligned} \Vert \dot{\boldsymbol{\epsilon }}|| = \xi s \cos (\xi x_1) Q \end{aligned}$$

with

11 $$\begin{aligned} s= \text {sgn} (\text {cos}(\xi x_1)), \quad Q= \sqrt{a_1^2 + \frac{1}{2} a_2^2 + \frac{1}{2} a_3^2} \end{aligned}$$

where $a_1$, $a_2$, $a_3$ are the velocity amplitude of the velocity field. Substituting Eq. (10) into Eq. (9) can obtain

12 $$\begin{aligned} \eta _{ij} \frac{d v_j}{d x_1} + b_i \xi s \cos (\xi x_1) Q + \lambda ^2 b_i \frac{d^2}{d x_1^2} \xi s \cos (\xi x_1) Q = 0, \quad i = 1, 2, 3 \end{aligned}$$

while it reduces to

13 $$\begin{aligned} \eta _{ij} a_j \xi \cos (\xi x_1) + b_i \xi s \cos (\xi x_1) Q + \lambda ^2 b_i \xi ^3 s \cos (\xi x_1) Q = 0, \quad i = 1, 2, 3 \end{aligned}$$

After then Eq. (8) reduces to

14 $$\begin{aligned} \eta _{ij} a_j + b_i (1-\lambda ^2 \xi ^2) Q = 0, \quad i = 1, 2, 3 \end{aligned}$$

where $\xi$ represents the wave length of the velocity field.

Given a stress state and a void ratio, i.e., two coefficients $\eta _{ij}$ and $b_{i}$ are fixed, $\xi$ can be derived by satisfying a non-zero solution of existing values of $a_1$, $a_2$, $a_3$ with

15 $$\begin{aligned} \eta _{ij} \alpha _j + b_i s (1- \lambda ^2 \xi ^2) = 0, \quad i = 1, 2, 3 \end{aligned}$$

where $\alpha _j= a_j/ Q$. Then the solution to Eq. (8) is derived as

16 $$\begin{aligned} \alpha _i = \frac{(1 - \lambda ^2 \xi ^2) \Lambda _i}{\Lambda _0}, \quad i = 1, 2, 3 \end{aligned}$$

where

17 $$\begin{aligned} \begin{aligned} \Lambda _0&= \det \begin{pmatrix} \eta _{11} & \eta _{12} & \eta _{13} \\ \eta _{21} & \eta _{22} & \eta _{23} \\ \eta _{31} & \eta _{32} & \eta _{33} \end{pmatrix}, \\ \Lambda _1&= \det \begin{pmatrix} -b_1 & \eta _{12} & \eta _{13} \\ -b_2 & \eta _{22} & \eta _{23} \\ -b_3 & \eta _{32} & \eta _{33} \end{pmatrix}, \\ \Lambda _2&= \det \begin{pmatrix} \eta _{11} & -b_1 & \eta _{13} \\ \eta _{21} & -b_2 & \eta _{23} \\ \eta _{31} & -b_3 & \eta _{33} \end{pmatrix}, \\ \Lambda _3&= \det \begin{pmatrix} \eta _{11} & \eta _{12} & -b_1 \\ \eta _{21} & \eta _{22} & -b_2 \\ \eta _{31} & \eta _{32} & -b_3 \end{pmatrix}. \end{aligned} \end{aligned}$$

As $\alpha _j$ must satisfy the condition:

18 $$\begin{aligned} \alpha _1^2 + \frac{1}{2}\alpha _2^2 + \frac{1}{2}\alpha _3^2 = 1 \end{aligned}$$

Combining Eq. (16) and Eq. (18), the wave length $\xi$ and the material parameter $\lambda$ to controlling shear band can be obtained

19 $$\begin{aligned} (1 - \lambda ^2 \xi ^2)^2 = \frac{\Lambda _0^2}{\Lambda _1^2 + \frac{1}{2} \Lambda _2^2 + \frac{1}{2} \Lambda _3^2} \end{aligned}$$

Real values of $\xi$ are defined by Eq. (19), and correspondingly, solutions $a_1$, $a_2$, $a_3$ to Eq. (14) exist if the condition is satisfied as

20 $$\begin{aligned} \Lambda _1^2 + \frac{1}{2}\Lambda _2^2 + \frac{1}{2}\Lambda _3^2 \ge \Lambda _0^2 \end{aligned}$$

The normalized inherent length is

21 $$\begin{aligned} \frac{l}{\lambda } = 2\pi \left( 1 - \sqrt{\frac{\Lambda _0^2}{\Lambda _1^2 + \frac{1}{2}\Lambda _2^2 + \frac{1}{2}\Lambda _3^2}} \right) ^{-1/2} \end{aligned}$$

### Shear band analysis for the non-gradient equation

The connection between shear band formation for the non-gradient theory (Eq. (1)) and the periodic solutions (Eq. (8)) for the gradient theory (Eq. (7)) is established. Unlike the linear analysis used for the elasto-plastic medium, the shear band analysis in the hypoplastic model incorporates an incremental nonlinear component (Eq. (1)), which leads to different solutions. To derive a criterion for the shear band formation, consider a velocity field, which depends on $x_1$, is continuous and undergoes a jump in its gradient on a plane $x_1$ = const. The condition for the formation of a shear band can be obtained as

22 $$ \begin{aligned} \llbracket {\dot{\bf{\sigma}}}_{1i} \rrbracket = 0, \quad i = 1, 2, 3 \end{aligned}$$

where $\llbracket \cdot \rrbracket$ denote a jump of the bracketed quantity across the discontinuity surface $x_1$ = const. Substituting Eq. (22) into Eq. (1), the jumps $\llbracket d v_j/ d x_1 \rrbracket$ can be obtained:

23 $$\begin{aligned} \eta _{ij} \llbracket \frac{d v_j}{d x_1} \rrbracket + b_i \llbracket \Vert \dot{\boldsymbol{\epsilon }} \Vert \rrbracket = 0, \quad i = 1, 2, 3 \end{aligned}$$

where $\eta _{ij}$ and $b_i$ represent the functions of the stress components and the void ratio, respectively, as the same with in Eq. (9). The solution of Eq. (23) can be written below if $\llbracket \Vert \dot{\boldsymbol{\epsilon }} \Vert \rrbracket$ is given:

24 $$\begin{aligned} \llbracket \frac{d v_j}{d x_1} \rrbracket = \llbracket \Vert \dot{\boldsymbol{\epsilon }} \Vert \rrbracket \frac{\Lambda _j}{\Lambda _0}, \quad j = 1, 2, 3 \end{aligned}$$

where $\Lambda _j$ and $\Lambda _0$ are listed in Eq. (17).

According to Wu [40], the jump of the velocity gradient across the discontinuity surface can be written as

25 $$\begin{aligned} \llbracket \frac{d v}{d x} \rrbracket = \left( \frac{d v}{d x} \right) ^{+} - \left( \frac{d v}{d x} \right) ^{-} \end{aligned}$$

where the superscripts + and − refer to values of a field quantity directly in front of and behind the discontinuity surface. Therefore, any pair of tensors $\dot{\boldsymbol{\epsilon }}^{+}$ and $\dot{\boldsymbol{\epsilon }}^{-}$ must satisfy

26 $$\begin{aligned} \Vert \llbracket \dot{\boldsymbol{\epsilon }} \rrbracket \Vert ^2 \ge \left( \Vert \dot{\boldsymbol{\epsilon }}^+ \Vert - \Vert \dot{\boldsymbol{\epsilon }}^- \Vert \right) ^2 \end{aligned}$$

where Eq. (26) can be written with satisfying condition in Eq. (25)

27 $$\begin{aligned} \llbracket \frac{d v_1}{d x_1} \rrbracket ^2 + \frac{1}{2} \llbracket \frac{d v_2}{d x_1} \rrbracket ^2 + \frac{1}{2} \llbracket \frac{d v_3}{d x_1} \rrbracket ^2 \ge \llbracket \Vert \dot{\boldsymbol{\epsilon }} \Vert \rrbracket ^2 \end{aligned}$$

Substituting Eq. (24) to Eq. (27) we can obtain

28 $$\begin{aligned} \Lambda _1^2 + \frac{1}{2}\Lambda _2^2 + \frac{1}{2}\Lambda _3^2 \ge \Lambda _0^2 \end{aligned}$$

This equation represents the requiring values of coefficients $\eta _{ij}$ and $b_i$ for the shear band analysis. We find that Eq. (28) is the same with Eq. (20). Therefore, an inherent length *l* exists for the strain-gradient hypoplastic model (Eq. (7)), allowing the existence of a periodic velocity field (Eq. (8)) only if the material is at a post-bifurcation state in the sense of the non-gradient hypoplastic theory. As inequalities Eqs. (20) and (28) become equalities, the inherent length at the point of bifurcation will reach to infinite value.

## One-component velocity analysis

If the kinematics is restricted to a one-component velocity field which depends on one spatial coordinate (e.g., undrained simple shear or one-dimensional compression-extension), the analyses of inherent length and shear band formation presented above can be performed in a similar way with the difference that the determinants degenerate into single stiffness coefficients, and the norm of $\dot{\boldsymbol{\epsilon }}$ becomes the absolute value of a strain rate component.

**Fig. 1:**
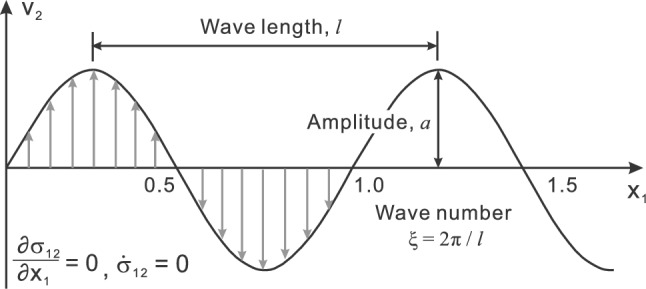
Schematic of the velocity field given in Eq. (29), while a small periodic perturbation $v_2(x_1) = a \sin (\xi x_1)$ imposed for the onset of localization

A simple shear condition is considered with a velocity field of $v_2 (x_1)$ applied to homogeneous state of granular material, which can be described by Eq. (1). The schematic illustration of this velocity field is shown in Fig. 1. Such deformation is physically relevant to the undrained shearing of a granular material saturated with an incompressible fluid. The velocity field for the inherent length analysis can be assumed as

29 $$\begin{aligned} v_2(x_1) = a \sin (\xi x_1), \quad \xi = \frac{2\pi }{l} \end{aligned}$$

where *a* is the velocity amplitude, and *l* is the wave length.

The condition of constant stresses expressed generally by Eq. (9) reduces now to a single condition $\dot{\boldsymbol{\sigma }}_{12}=0$:

30 $$\begin{aligned} \eta _{22} \frac{dv_2}{dx_1} + \frac{1}{\sqrt{2}} b_2 \left| \frac{dv_2}{dx_1} \right| + \frac{1}{\sqrt{2}} \lambda ^2 b_2 \frac{d^2 }{dx_1^2} \left| \frac{dv_2}{dx_1} \right| = 0 \end{aligned}$$

As $s= \text {sgn} (\text {cos}(\xi x_1))$, substituting Eq. (29) to Eq. (30) can obtain

31 $$\begin{aligned} \eta _{22} a \xi \cos (\xi x_1) + \frac{1}{\sqrt{2}} b_2 s \left| a\right| \xi \cos (\xi x_1) - \frac{1}{\sqrt{2}} \lambda ^2 b_2 s \left| a\right| \xi ^3 \cos (\xi x_1) = 0 \end{aligned}$$

Then reduces to:

32 $$\begin{aligned} \eta _{22}a+ \frac{1}{\sqrt{2}}b_2s\left| a \right| (1-\lambda ^2\xi ^2) = 0 \end{aligned}$$

since $a/\left| a \right| = \pm 1$, it follows that a non-zero solution *a* to Eq. (32) exists if and only if

33 $$\begin{aligned} \left| \frac{b_2 s (1 - \lambda ^2 \xi ^2)}{\sqrt{2} \eta _{22} } \right| = 1 \end{aligned}$$

it can be written as

34 $$\begin{aligned} 1 - \lambda ^2 \xi ^2 = \sqrt{2} \left| \frac{ \eta _{22}}{ b_2}\right| \end{aligned}$$

where with Eq. (29), it can be reduced to

35 $$\begin{aligned} \lambda \frac{2\pi }{l} = \left( 1 - \sqrt{2} \left| \frac{ \eta _{22}}{ b_2}\right| \right) ^{1/2} \end{aligned}$$

where it can be normalized with the possible wave length *l*

36 $$\begin{aligned} \frac{l}{\lambda } = 2\pi \left( 1 - \sqrt{2} \left| \frac{\eta _{22}}{b_2} \right| \right) ^{-1/2} \end{aligned}$$

where if a real wave length and a velocity filed as Eq. (29) exist

37 $$\begin{aligned} \frac{1}{\sqrt{2}} \left| \frac{ \eta _{22}}{ b_2}\right| > 1 \end{aligned}$$

which solution given in Eq. (36) is the same presented by Osinov and Wu [23]. It can be seen the inherent length of shear band under simple shear condition is the same whether strain-gradient is extended in linear or nonlinear tensorial functions of hypoplastic model.

For a velocity field $v_2(x_1)$, the stress increment tensor of the component ${\sigma }_{12}$ of hypoplastic model without the strain-gradient extension can be written as

38 $$\begin{aligned} \dot{{\sigma }}_{12}=\eta _{22} \frac{\partial v_2}{\partial x_1} + \frac{1}{\sqrt{2}} b_2 \left| \frac{\partial v_2}{\partial x_1} \right| \end{aligned}$$

Note that the assumption given in Eq. (37) will result in negative stiffness of ${\sigma }_{12}$ in Eq. (38) no matter whether the values of strain rate $\frac{\partial v_{2}}{\partial x_{1}}$ are positive or negative. Therefore, solutions of Eq. (38) are possible if and only if the stress state lies on the descending branch of the stress–strain diagram for simple shear for the non-gradient constitutive relation. According to Osinov and Wu [23], at the point of maximum shear stress where the stiffness is zero, $l\rightarrow \infty$. In other words, the granular material under simple shear conditions must undergo a post-peak stage, i.e., as localization occurs, for $\boldsymbol{\sigma }_{12}$ to have meaningful solutions. This is when gradient-enhanced models are necessary to predict stable, structured deformation patterns of post-localization regime.

## Hypoplastic model for granular materials

A basic hypoplastic model proposed by Wu et al. [42] has demonstrated its outstanding performance of describing the mechanical behaviors of sand. Compared with the constitutive relationship used by Von Wolffersdorff [30], it requires less parameters and consists of 3 linear terms and 1 nonlinear term. In this work, we adopt a recently proposed hypoplastic model for granular materials (Simhypo-sand) by Wang et al. [34] for the strain-gradient extension, as follows:

39 $$\begin{aligned} \mathring{\boldsymbol{\sigma }}=f_s\Big [\textrm{tr}(\boldsymbol{\sigma })\dot{\boldsymbol{\epsilon }}+ f_v\textrm{tr}(\dot{\boldsymbol{\epsilon }})\boldsymbol{\sigma }+ {a_h}^2\frac{\textrm{tr}(\boldsymbol{\sigma }\dot{\boldsymbol{\epsilon }})}{\textrm{tr}(\boldsymbol{\sigma })}\boldsymbol{\sigma }+ f_da_h(\boldsymbol{\sigma }+\boldsymbol{\sigma }^{*}) (\left\| \dot{\boldsymbol{\epsilon }} \right\| + \lambda ^2\nabla ^2 \left\| \dot{\boldsymbol{\epsilon }} \right\| ) \Big ] \end{aligned}$$

where $f_s$ and $f_v$ are multipliers accounting for the stiffness and volumetric response of the materials, respectively.

40 $$\begin{aligned} f_s= -\frac{E_i}{3(1+\nu _i)\sigma _c},\; f_v= \frac{3\nu _i+a_h\sqrt{1+2\nu _i^2}}{1-2\nu _i} -\frac{{a_h}^2}{3} \end{aligned}$$

in which $E_i$ and $\nu _i$ are the material parameters. $\sigma _c =100$ kPa is used for material calibration.

The factor $f_d$ is a density function, which allows this model to consider impact of variation in the solid fraction during shearing [39]. In this work, the following simple function is adopted:

41 $$\begin{aligned} f_d=\left( \frac{e-e_\text {d}}{e_\text {c}-e_\text {d}}\right) ^{\alpha} \end{aligned}$$

where *e*, $e_{d}$ and $e_{c}$ are the current, minimum, and critical state void ratios; $\alpha$ is a constant that controls the degree of strain softening [13, 31, 32]. The logarithmic critical state line proposed by Li and Wang [16] is adopted. The function of critical state void ratio $e_{c}$ dependent on the stress level is adopted as:

42 $$\begin{aligned} e_{c}=e_{\Gamma } \textrm{exp}\Big [{-\zeta \big (\frac{p'}{p_a}}\big )^{\xi_h }\Big ] \end{aligned}$$

where $e_{\Gamma }$, $\zeta$, and $\xi_h$ are material parameters for characterizing the critical state of granular materials. $p'$ is the effective mean stress and $p_a$ is the atmospheric pressure for normalization. The evaluation of void ratios at current and critical states give rise to different values of $f_d$, which is less than 1 for a dense state, greater than 1 for a loose state, and equals 1 at the critical state [34].

In this model, the parameter *a*_*h*_ is related to the critical state value of the normalized deviatoric stress $\Vert \boldsymbol{\sigma }_c^* \Vert /\textrm{tr}(\boldsymbol{\sigma }_c)$, given:

43 $$\begin{aligned} a_h = \frac{\eta \sqrt{3}(3-\text {sin}\phi _c)}{2\sqrt{2}\text {sin}\phi _c}\; \text {with } \;\eta = \frac{2I_1}{3\sqrt{I_1^2-3I_2}\sqrt{(I_1I_2-I_3)/(1_1I_2-9I_3)-1}} \end{aligned}$$

in which $\phi _c$ is the critical state friction angle and the factor η is adopted to incorporate the SMP failure criterion. Clearly, the size of the yield surface evolutes with the variation of the density function $f_d$ before the material reaching the critical state ($f_d=1$ at the critical state). This suggests that the threshold yield stress for starting flow is also related to the initial condition, i.e., void ratio, of the granular material.

This hypoplastic model only contains seven parameters: $E_i$, $v_i$, $\phi _c$, and $\alpha$ for the hypoplastic model, $e_\Gamma$, $\zeta$, and $\xi_h$ for the critical state of granular materials. Most of the parameters can be obtained from triaxial compression tests, as detailed in the literature [8, 9, 34].

## Simple shear tests

In this section, the shear band formation under simple shear conditions is adopted. We assume that the dimension of the soil layer is much larger than the thickness of the emerging shear band. We consider a boundary condition of shear band induced in a thin zone with initially lower stiffness, which is obtained by prescribing a weak inhomogeneity point in the void ratio profile.

Consider the strain-controlled simple shearing of a layer between $x_1$ = 0 and $x_1$ = *L* with prescribed velocities at the boundaries:

44 $$\begin{aligned} v_2(0, t) = 0,\quad v_2(L, t) > 0 \end{aligned}$$

The equilibrium equation for $\sigma _{12}$ is

45 $$\begin{aligned} \frac{\partial \sigma _{12}}{\partial x_1} = 0 \end{aligned}$$

after the time differentiation reduces to an ordinary differential equation for the current velocity field:

46 $$\begin{aligned} \frac{d}{dx_1}\left( \eta _{22} \frac{dv_2}{dx_1} + \frac{1}{\sqrt{2}} b_2 \left| \frac{dv_2}{dx_1} \right| + \frac{1}{\sqrt{2}} \lambda ^2 b_2 \frac{d^2 }{dx_1^2} \left| \frac{dv_2}{dx_1} \right| \right) = 0 \end{aligned}$$

The fourth-order derivative will require two boundary conditions to solve Eq. (44):

47 $$\begin{aligned} \left. \frac{d^2 v_2}{d x_1^2} \right| _{(0, t)} = 0, \quad \left. \frac{d^2 v_2}{d x_1^2} \right| _{(L, t)} = 0 \end{aligned}$$

where these two boundary conditions ensure the homogeneous deformation far away from the shear band where the stress state and the void ratio are homogeneous. Meanwhile, this higher-order gradient equation produces a linear velocity field if the soil layer is initially homogeneous, which the solution is the same as that of the non-gradient equation. The average shear deformation in the layer will be denoted by $\gamma$:

48 $$\begin{aligned} \gamma = \frac{\partial u_2}{\partial x_1}= \frac{u_2(L) - u_2(0)}{L} \end{aligned}$$

where $u_2(x_1)$ is the displacement in the $x_2$-direction.

### homogeneous sample

Classic examples of simple shear tests are conducted, whereas Osinov and Wu [23] assume the same conditions for pure shear tests. The material parameter is listed in Table 1. The initial stress state in all examples below is taken to be spatially homogeneous: $\sigma _{11}$= 100 kPa, $\sigma _{22}$= 50 kPa, $\sigma _{33}$= 70 kPa, $\sigma _{12}$= $\sigma _{13}$= $\sigma _{23}$= 0.

**Table 1:** Material parameters for simulating simple shear tests

Parameter	$E_i$ (MPa)	$\phi _c$	$\nu _i$	$e_d$	$e_{\Gamma }$	$\zeta$	$\xi_h$	$\alpha$
Value	13.0	$30.0^{\circ }$	0.28	0.55	0.95	0.102	0.107	0.3

**Fig. 2:**
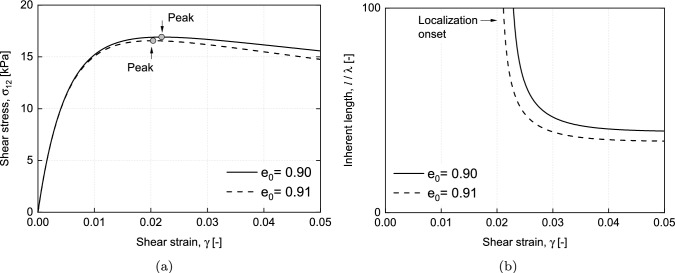
Simple shear calculated for a homogeneous sample with different initial void ratios. (a) Shear stress ($\sigma _{12}$) vs. Shear strain ($\gamma$). (b) inherent length ($l/\lambda$) of shear band derived by Eq. (36) vs. shear strain ($\gamma$)

The simulation results of the simple shear test indicate that small variations in the initial void ratio ($e_0$), representing the initial state of the granular material, influence the mechanical response of the homogeneous sample. As shown in Fig. 2a, the stress–strain curves reveal that the looser material ($e_0$ = 0.91) exhibits a lower shear stress and a more pronounced descending branch compared to the denser sample ($e_0$ = 0.90). Figure 2b presents the normalized inherent length derived from Eq. (36) based on the nonlinear strain-gradient extension of hypoplastic model. It is found that the inherent length does not exist at the shear beginning, it shows an infinite trend at the extremum point. At the post-localization regime, the value of inherent length gradually shows constant trend. In addition, the shear band begin to form immediately after the peak, denoting the onset of localization.

### Inhomogeneous cases

**Fig. 3:**
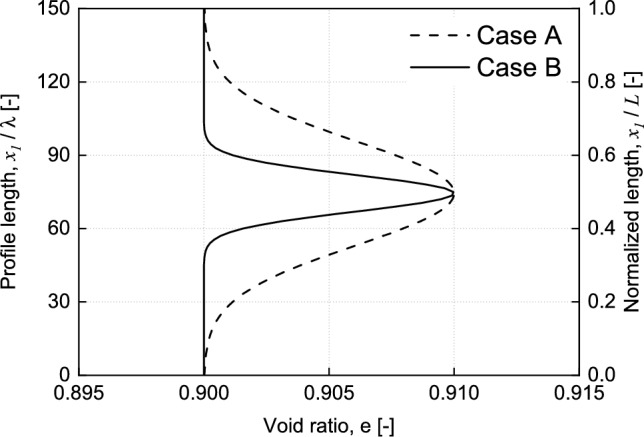
Inhomogeneous distribution of the void ratio with a weak point used for the simulations

As shown in Fig. 3, numerical simulation of shear band analysis will be carried out on two representative cases A and B of an inhomogeneous sample with different initial void ratio distributions. The localized peak in void ratio (the weak point at $e_0$= 0.91) represents an initially looser zone. Case A shows a more diffuse profile of the granular material, whereas Case B represents a sharper, more localized change in the initial void ratio distribution.

**Fig. 4:**
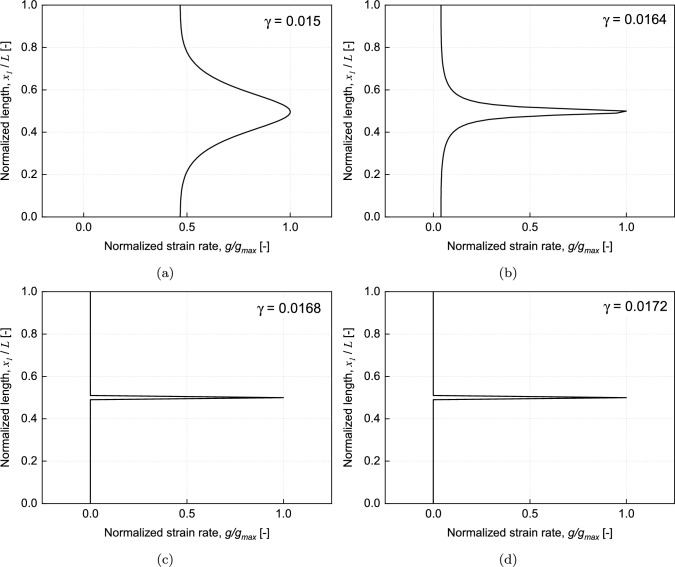
Normalized strain rate $g = \partial v_2/\partial x_1$ in an inhomogeneous sample (Case A in Fig. 3) at different stages of the shearing calculated with the non-gradient model in Eq. (1)

The simulation results of the simple shear test using the non-gradient hypoplastic model are shown in Fig. 4. The development of the shear band is illustrated by the normalized strain rate, $g = \partial v_2 / \partial x_1$, plotted along the normalized profile length, $x_1 / L$. The results indicate that the spatial distribution of strain rate is strongly influenced by the initial void ratio distribution. As shear progresses toward the peak of the stress–strain curve, recalled in Fig. 2a, deformation becomes increasingly localized within a narrow zone. This localization manifests as a concentration of strain rate near the initially weaker (looser) region. The resulting profile resembles a Dirac delta-like distribution, which sharpens rapidly within a very small range of shear strain and collapses into an infinitely thin localization zone. Beyond this point (in our case is $\gamma$= 0.0172), the mathematical model ceases to yield a valid solution.

**Fig. 5:**
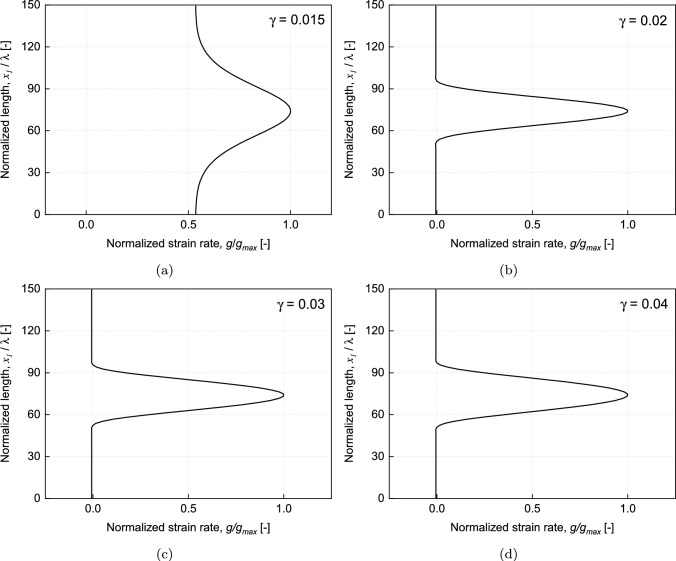
Normalized strain rate $g = \partial v_2/\partial x_1$ in an inhomogeneous sample (Case A in Fig. 3) at different shear strains calculated with the gradient model by Eq. (7)

**Fig. 6:**
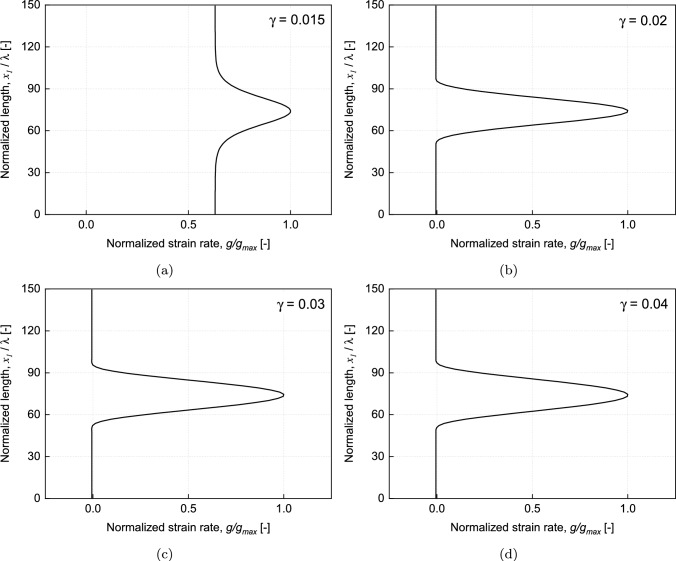
Normalized strain rate $g = \partial v_2/\partial x_1$ in an inhomogeneous sample (Case B in Fig. 3) at different shear strains calculated with the gradient model by Eq. (7)

The simulation results of Cases A and B, obtained using the gradient model by Eq. (7), are shown in Figs. 5 and 6, respectively. Compared to the results obtained from the non-gradient model (Eq. (1)), the strain-gradient extension of the hypoplastic model predicts the formation of a shear zone before the peak of the stress–strain curve. In the post-localization regime, numerical results show that strain rates outside the shear zone remain non-zero and slightly negative, indicating material undergoes unloading, consistent with observations reported by Osinov and Wu [23].

**Fig. 7:**
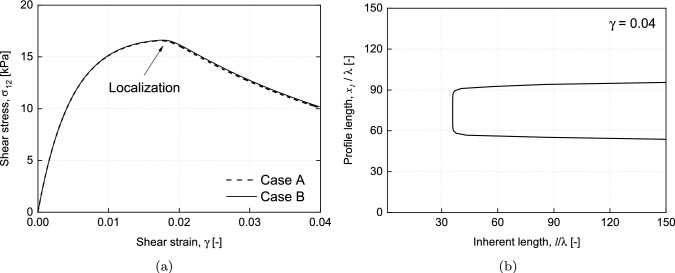
Simple shear tests for the inhomogeneous samples calculated with the gradient model. **a** Shear stress ($\sigma _{12}$) versus shear strain ($\gamma$) derived by Eq. (7). **b** inherent length ($l/\lambda$) of shear band derived by Eq. (36) for Case A

As shown in Fig. 7a, the stress–strain curve of the inhomogeneous sample obtained by the gradient model (Eq. (7)) shows an obvious post-peak trend. The descending branch indicates the onset of localization. Moreover, the gradient model introduces an inherent length scale associated with the shear band, independent of the initial void ratio distribution. This characteristic length, interpreted as the shear band thickness, is governed by the parameter $\lambda$, which is determined solely by the intrinsic properties of the granular material. The distribution of the inherent length for case A is shown in Fig. 7b at $\gamma$= 0.04 as post-localization regime. The values of the inherent length at each point along the material profile was calculated by Eq. 36. Accordingly, the shear band thickness normalized by $\lambda$ is approximately equal to 40.

## Discussions

**Fig. 8:**
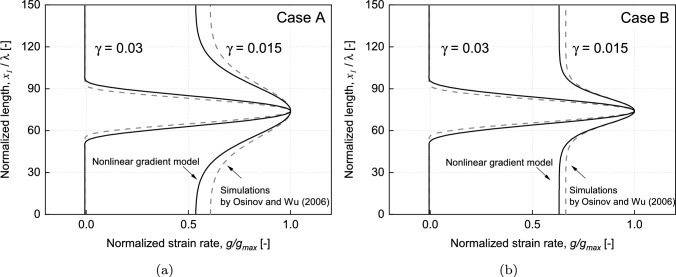
Comparison of the simple shear tests using the gradient nonlinear model in this study and the pure shear test using linear gradient extension by Osinov and Wu [23]. **a** Case A. **b** Case B

The model performance is compared with the previous work by Osinov and Wu [23], which strain-gradient extension was implemented on hypoplastic framework of Von Wolffersdorff [30]. The numerical results solved by two models of the representative inhomogeneous cases (Fig. 3) are shown in Fig. 8. The extended model in the present study and the linear gradient model proposed by Osinov and Wu [23] both capture the inherent length scale of shear band at post-localization regime, which are not influenced by the initial distributions of void ratio. Compared to the linear gradient model, the nonlinear gradient model results in faster localization of strain into the shear band, as evidenced by the lower strain rate outside the band at the same shearing stage. This behavior may be attributed to differences in the underlying constitutive models. Nevertheless, the extended model still produces localization behavior that is comparable to the numerical results reported by Osinov and Wu [23]. It is noticed that the shear band thickness predicted by nonlinear gradient model is slightly broader, whereas it still comparable to the previous predictions.

A key improvement of our extended model lies in its treatment of material rotation and objectivity. According to Osinov and Wu [23], the linear gradient extension of the hypoplastic model has:

49 $$\begin{aligned} \mathring{\boldsymbol{\sigma }}= {\mathcal {L}}(\boldsymbol{\sigma } ):(\dot{\boldsymbol{\epsilon }} - \lambda ^2\nabla ^2\dot{\boldsymbol{\epsilon }}) + {{\textbf {N}}}(\boldsymbol{\sigma })\left\| \dot{\boldsymbol{\epsilon }} \right\| \end{aligned}$$

**Fig. 9:**
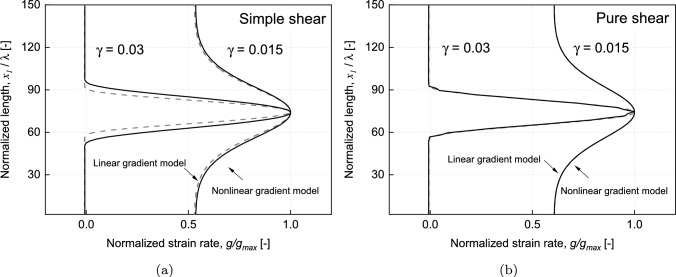
Comparison of gradient extensions applied to different hypoplastic models. **a** Simple shear test using the gradient extension introduced in Eq. (39). **b** Pure shear test using the gradient extension proposed by Osinov and Wu [23], applied to model of Von Wolffersdorff [30]

For a better comparison, both linear and nonlinear gradient extensions are applied to two different hypoplastic models, namely Eq. (39) and the model proposed by Von Wolffersdorff [30], which was adopted by Osinov and Wu [23]. As shown in Fig. 9, the general localization patterns and shear band thicknesses remain comparable between the linear and nonlinear versions of each model. Theoretically, gradient extensions of both the linear or nonlinear terms of the hypoplastic model lead to the consistent results of shear band development. Compared with the gradient extension applied to the linear term, our extension of the nonlinear term provides a more accurate representation of the mechanical response of granular materials. The nonlinear term primarily governs the state-dependent behavior and ensures rate independence through the strain rate norm, making this approach advantageous for numerical modeling of shear banding.

Note that for the linear gradient extension in Eq. (49), it is necessary to assume that, under pure shear conditions, objectivity is maintained by adopting a modified Jaumann stress rate that includes both the spin tensor and its Laplacian:

50 $$\begin{aligned} \mathring{\boldsymbol{\sigma }} = \dot{\boldsymbol{\sigma }} + \boldsymbol{\sigma } \left( \dot{\bf{\omega}}- \lambda ^2 \nabla ^2 \dot{\bf{\omega}} \right) - \left( \dot{\bf{\omega}}- \lambda ^2 \nabla ^2 \dot{\bf{\omega}} \right) \boldsymbol{\sigma } \end{aligned}$$

While this correction ensures frame invariance, it introduces additional mathematical complexity and implementation effort. In contrast, the nonlinear gradient extension of hypoplastic model avoids these complications by regularizing the post-localization response through the scalar norm of the strain rate and its Laplacian. This approach captures rotational effects without requiring spin-related corrections, resulting in a simpler and physically consistent model. In addition, this improvement is appropriate to analyzing realistic shear band problems involving rotational deformation, such as those occurring in granular materials under simple shear conditions.

## Conclusions

Conventional continuum models are limited in their ability to capture shear band formation due to the absence of inherent length scales. This study introduces a strain-gradient extension of the hypoplastic model by incorporating a length scale parameter into the nonlinear tensorial function. The extended model successfully captures shear band behavior in granular materials in the post-localization regime while preserving the conciseness of the hypoplastic framework. Numerical simulations of simple shear tests on two inhomogeneous samples demonstrate that the original non-gradient model produces infinitely thin shear bands and fails to sustain large shear strains. In contrast, the extended model predicts finite-thickness shear bands, governed by inherent material length rather than the initial distribution of void ratio.

Compared to the linear gradient model in previous work [23], the nonlinear gradient model exhibits lower strain rates at the boundaries of the shear band in the early stages of localization, while maintaining comparable shear band thicknesses. Due to the extension in the rotation-invariant scalar quantity, i.e., the norm of the strain rate, the extended model naturally accommodates simple shear deformation without requiring explicit spin tensor corrections. This makes it particularly well-suited for realistic simulations of strain localization in granular materials.

## Data Availability

The data that support the findings of this study are available from the corresponding author upon reasonable request.

## Strain gradient extension on Simhypo-sand model

The strain-gradient extension of the Simhypo-sand model by Wang et al. [34] is given as:

A.1 $$\begin{aligned} \mathring{\boldsymbol{\sigma }}=f_s\Big [\textrm{tr}(\boldsymbol{\sigma })\dot{\boldsymbol{\epsilon }}+ f_v\textrm{tr}(\dot{\boldsymbol{\epsilon }})\boldsymbol{\sigma }+ {a_h}^2\frac{\textrm{tr}(\boldsymbol{\sigma }\dot{\boldsymbol{\epsilon }})}{\textrm{tr}(\boldsymbol{\sigma })}\boldsymbol{\sigma }+ f_da_h(\boldsymbol{\sigma }+\boldsymbol{\sigma }^{*}) (\left\| \dot{\boldsymbol{\epsilon }} \right\| + \lambda ^2\nabla ^2 \left\| \dot{\boldsymbol{\epsilon }} \right\| ) \Big ] \end{aligned}$$

where $\lambda$ is a material parameter which has length scale for strain-gradient extension.

Multipliers $f_s$ and $f_v$ accounts for the stiffness and volumetric response of the materials, respectively:

A.2 $$\begin{aligned} f_s= -\frac{E_i}{3(1+\nu _i)\sigma _c},\; f_v= \frac{3\nu _i+f_da_h\sqrt{1+2\nu _i^2}}{1-2\nu _i} -\frac{{a_h}^2}{3} \end{aligned}$$

in which $E_i$ and $\nu _i$ are material parameters. $\sigma _c =100$ kPa is used for material calibration.

The density factor $f_d$ is given as follows:

A.3 $$\begin{aligned} f_d=\left( \frac{e-e_\text {d}}{e_\text {c}-e_\text {d}}\right) ^\alpha \end{aligned}$$

where $\alpha$ is a constant that controls the degree of strain softening; *e* and $e_{d}$ are the current and minimum void ratios, $e_{c}$ is the critical state void ratio:

A.4 $$\begin{aligned} e_{c}=e_{\Gamma } \textrm{exp}\Big [{-\zeta \big (\frac{p'}{p_a}}\big )^{\xi_h }\Big ] \end{aligned}$$

where $e_\Gamma$, $\zeta$, and $\xi_h$ are material parameters for characterizing the critical state of granular materials. $p^\prime$ is the effective mean stress and $p_\text {a}$ = −101.325 kPa is the atmospheric pressure for normalization.

The factor *a*_*h*_ regulates the strength of the material through:

A.5 $$\begin{aligned} a_h = \frac{\eta \sqrt{3}(3-\text {sin}\phi _c)}{2\sqrt{2}\text {sin}\phi _c}\; \text {with } \;\eta = \frac{2I_1}{3\sqrt{I_1^2-3I_2}\sqrt{(I_1I_2-I_3)/(1_1I_2-9I_3)-1}} \end{aligned}$$

where $\phi _c$ is the critical state friction angle and the factor $\eta$ is adopted to incorporate the SMP failure criterion.

There are eight parameters for the proposed model: $E_i$, $\nu _i$, $\phi _c$, $\alpha$, $e_\Gamma$, $\zeta$, $\xi_h$, and $\lambda$. $E_i$ (initial elastic modulus), $\nu _i$ (initial Poisson’s ratio), and $\phi _c$ (critical state friction angle) can be obtained from a single triaxial compression test with confining pressure of 100 kPa. $\alpha$ can be determined by trial and error based on the density effect on strain softening or hardening. The critical state parameters $e_{\Gamma }$, $\zeta$, and $\xi_h$ can be measured from an $e-\text {log}(p')$ plot of critical states based on three triaxial compression tests. $\lambda$ is the inherent length scale depending on the mean grain size of the material. In addition, the constitutive model requires the initial value of void ratio.
